# News and Events of the Week

**Published:** 1911-05-27

**Authors:** 


### News and Events of the Week.

Dr. Clauston and Dr. G. M. Robertson, of Edinburgh, Dr. Henry Rayner, of London, and Dr. Urquhart, of Perth, have been elected corresponding members of the Société de Psychiatrie of Paris.

Messrs. P. S. King and Son will shortly issue "Medical Revolution: A Plea for National Prevention on the Basis of a Natural Interpretation of Disease." Now that legislation for the improvement of the national health is imminent, and a great conference on destitution is about to meet, this book will merit the attention of all who have the national well-being at heart.

A record has once more been achieved by "Printers' Pie," 1911, which is obtainable at all bookstalls at the usual price, 1s. This evergreen annual, edited by Mr. W. Hugh Spottiswoode, has always had the distinction of being sold out by the publisher in advance of publication, but this year's volume has been sold out by the publisher a full fortnight ahead of its appearance, and, from the orders still coming in, it is likely that the demand will be almost double that of last year. Mr. Spottiswoode's aim this year has been to still further extend the lightness and brightness of the issue, and the coloured illustrations and prose and verse contributions will represent the last word in modern humour.

A great step in advance is to be taken with regard to the regulation of the milk-trade in Brussels. At the demand of the Conference of Burgomasters of the Brussels district, the "Commission Permanente du Lait" has just issued the text of a Bill to regulate the production, transport, and sale of milk destined for the capital and its suburbs. This project, which will be submitted to the various communal councils, is still imperfect, but it constitutes a step in the right direction towards proper control. In the opinion of the Commission, it will not be able to be enforced until such time as the Royal decrees with reference to the milk-trade have been modified, but the Government would seem well disposed towards the necessary alterations.

The Royal Geographical Society has this year bestowed the "Back Bequest" on the senior C.M.S. Punjab Medical missionary, Dr. Arthur Neve, who addressed an enthusiastic meeting at the Queen's Hall on Friday, last week. Dr. Neve, who has seen nearly thirty years' service, had come "straight from the front." He began by sending two or three blind men to the Quetta Mission Hospital, and paid their expenses. These men went back with recovered sight. "A few days later," Dr. Neve added, "I received a wire from Dr. Henry Holland: 'Come and help me. Two hundred out-patients a day, and heaps of cataract cases.' I joined him, and we had enormous crowds —four or five hundred patients being seen every day. One of us was hard at work all day on cataract, and in three weeks we had about 300 cataract cases! The news of this spread far and wide, and much was written about it, even in disloyal papers. Trains brought cataract cases from all over the place. Now an Hindoo banker has built an eye hospital costing some thousands of pounds, and when it was opened recently all the leading men of the place spoke warmly of the work of the medical missionaries."

The University College Committee of London University report the receipt from Mr. Carnegie of £5,000 towards the building and equipment of the Medical Sciences Institute of the College, the Physiology Section of which was opened two years ago.

Mr. G. A. Hutchinson, referring to the fact that the King and Queen and the Queen Mother had been patrons of the great Nautical Mission, said Queen Mary had also given them permission to name their next hospital ship, "The Queen Mary." (Cheers.)

Mr. Finlay Smith, of Gadgirth, Ayrshire, lately a member of the firm of tobacco manufacturers, aged eighty-one, left personal estate in the United Kingdom valued at £552,414, and has bequeathed £2,000 each to the Royal Infirmary, the Western Infirmary, the Victoria Infirmary, all of Glasgow.

At the recent meeting of the Senate of London University the Academic Council reported an offer from Sir Felix Semon, M.D., to transfer to the University, for the foundation of a Lectureship in Laryngology, a sum of money amounting to £1,040 presented to him by the British laryngologists on his retirement from practice. The benefaction was accepted upon the conditions offered.

We have received the second issue for 1911 of "The Public Health Service Directory and Year-Book" (Hodgetts, Ltd., 36-38 Whitefriars Street, E.C., 7s. 6d. net, post free). It is compiled by the Editor of "The Medical Officer," and contains a mass of detail concerning the aims and administration of the Public Health Service. Sanitary and school medical inspection all over the United Kingdom is dealt with at length. The books is also a Who's Who of medical officers, public analysts, veterinary officers, sanitary inspectors, inspectors of medicines, school nurses, and matrons of isolation hospitals.

Sir Alfred Pearce Gould, K.C.V.O. (President of the Clinical Section of the Royal Society of Medicine) presiding at the Thirtieth Annual Meeting of the Royal National Mission to Deep Sea Fishermen, held at Queen's Hall, on Friday last, referred to the medical part of the work. He said that society gave the injured and sick fishermen the best help it could get. Not only were they provided with highly qualified doctors, but with well equipped hospitals and hospital ships. The society did its very utmost to help those men in their physical necessities. Sir Alfred was elected a member of the Council most enthusiastically, and afterwards appointed Chairman of the Hospital Committee.

Burdett's Hospitals and Charities for 1911 is now ready. (The Scientific Press, 28 and 29 Southampton Street, Strand, price 10s. 6d. net). This ideal work of reference contains special new chapters on the Voluntary Hospitals in 1910; Soundness of the Voluntary System; The need of greater zeal on the part of those who accept office; State-aided Hospitals in the U.S.A. and Canada; The Appropriation of Public Funds for the partial support of Voluntary Hospitals in U.S.A. and Canada; The Need of an Apostle and of greater interest on the part of the Clergy, etc., etc.

